# Randomized scheduling feasibility study of S-1 for adjuvant chemotherapy in advanced head and neck cancer

**DOI:** 10.1038/sj.bjc.6602804

**Published:** 2005-09-27

**Authors:** M Tsukuda, A Kida, M Fujii, N Kono, T Yoshihara, Y Hasegawa, M Sugita, and Chemotherapy Study Group of Head and Neck Cancer

**Affiliations:** 1 Department of Biology and Function in the Head and Neck, Yokohama City University Graduate School of Medicine, Kanazawa-ku, Yokohama, Japan; 2 Department of Otorhinolaryngology – Head and Neck Surgery, Nihon University School of Medicine, Itabashi-ku, Tokyo, Japan; 3 Division of Hearing and Balance Research, National Institute of Sensory Organs, National Tokyo Medical Center, Meguro-ku, Tokyo, Japan; 4 Department of Otorhinolaryngology – Head and Neck Surgery, Kyorin University School of Medicine, Mitaka-City, Tokyo, Japan; 5 Department of Otolaryngology, Tokyo Women's Medical University, Shinjyuku-ku, Tokyo, Japan; 6 Department of Head and Neck Surgery, Aichi Cancer Center Hospital, Chikusa-ku, Nagoya, Japan; 7 Department of Environmental and Occupational Health, Toho University School of Medicine, Ota-ku, Tokyo, Japan

**Keywords:** advanced head and neck cancer, S-1, adjuvant chemotherapy, feasibility study

## Abstract

The purpose of this study was to determine the feasible adjuvant therapy administration schedule of S-1 for locoregionally advanced squamous cell carcinoma of the head and neck (SCCHN). Patients receiving definitive treatments were randomly assigned to either arm A (51 cases) receiving oral S-1 of 2-week administration followed by 1-week rest for 6 months, or arm B receiving S-1 of 4-week administration followed by 2-week rest for 6 months. Planned treatment was given in 40% of patients in arm A and 29% in arm B. The cumulative rates of the relative total administration dose of S-1 at 100% were 54.9% (95% CI: 40.1–69.7%) in arm A and 34.3% (95% CI: 21.1–47.4%) in arm B, respectively (*P*=0.054). Adverse events were recorded in 41 patients (82.0%) in arm A and 48 patients (94.1%) in arm B (*P*=0.060). The incidences of diarrhoea (10 *vs* 28%; *P*<0.05) and skin toxicities (18 *vs* 37%; *P*<0.05) were significantly higher in arm B. One-year disease-free survival was similar in both arms: arm A 81.2% (95% CI: 70.0–92.4%); arm B 77.0% (95% CI: 65.0–89.0%). The schedule of 2-week administration followed by 1-week rest seems to be more feasible for oral 6-month administration of S-1 in adjuvant chemotherapy of locoregionally advanced SCCHN.

Squamous cell carcinoma of the head and neck (SCCHN) is a major public health problem because of the poor outcome (Landis *et al*, 1999). Two-thirds of patients present with locally advanced lesions (T3 or T4) and/or regional lymph node involvement (N1–N3). Recently, many studies on locoregionally advanced SCCHN have been focusing on neoadjuvant chemotherapy (NAC) with a high complete response (CR) rate followed by definitive radiotherapy, or concurrent chemoradiotherapy showing to improve the survival rate and to preserve functions and organs (Pignon *et al*, 2000; Monnerat *et al*, 2002; Forastiere *et al*, 2003; Haddad *et al*, 2003). However, 20–30% of advanced patients with a pathologically CR even after the recent aggressive treatment has been showing locoregionally recurrent and/or distant metastatic tumours (unpublished data in our institutes) resulting in the poor outcome nevertheless (Argiris *et al*, 2004; Brockstein *et al*, 2004; Cohen *et al*, 2004). Based on these results, more advanced multidisciplinary treatments should be indispensable in the aim of improving the poor outcome of patients with advanced SCCHN. Adjuvant chemotherapy might be a one of the candidates. Several randomised trials of adjuvant (maintenance) chemotherapy in advanced SCCHN have been done (Ervin *et al*, 1987; Jacobs and Makuch, 1990; Laramore *et al*, 1992; Johnson *et al*, 1996). A few randomised trials regarding adjuvant chemotherapy reported the possibility of survival benefits (Ervin *et al*, 1987; Johnson *et al*, 1996). Instead of intravenous administration of cisplatin (CDDP) and/or other agents, oral anticancer drugs are attractive for outpatient use as adjuvant chemotherapy. The fluoropyrimidine anticancer agent 5-fluorouracil (5-FU) is active in a variety of solid tumours, particularly gastric, colorectal and SCCHN. Recently, the adjuvant chemotherapy of UFT (tegafur and uracil in a 1 : 4 molar concentration), one of the oral 5-FU agents, has shown a significant survival benefit in a wide range of carcinomas (Akasu *et al*, 2004; Kato *et al*, 2004; Kinoshita *et al*, 2005; Noguchi *et al*, 2005). Furthermore, a prospective randomized trial to evaluate the efficacy of adjuvant chemotherapy in SCCHN from 67 institutions in Japan showed the 1-year administration of UFT (300 mg day^−1^) in cases receiving curative surgical treatment was clarified to prevent distant metastasis significantly with a small survival benefit compared to the outcome of the control cases receiving curative surgery without adjuvant chemotherapy of UFT (Tsukuda *et al*, 1994).

S-1 (Taiho Pharmaceutical Co., Ltd, Tokyo, Japan) is a dihydropyrimidine dehydrogenase (DPD)-inhibitory fluoropyrimidine (DIF), which showed the highest response rate among many oral anticancer agents against unresectable advanced carcinomas in phase II studies (Schoffski, 2004). S-1 is an oral anticancer agent comprised of tegafur, 5-chloro-2, 4-dihydroxypyridine, and potassium oxonate, in a molar ratio of 1 : 0.4 : 1 (Shirasaka *et al*, 1996a, 1996b; Schoffski, 2004). Tegafur is a prodrug of 5-FU and 5-Chloro-2, 4-dihydroxypyridine enhances the serum 5-FU concentration by the competitive inhibition of DPD, an enzyme responsible for 5-FU catabolism. Potassium oxonate, a reversible competitive inhibitor of orotate phosphoribosyl transferase, inhibits phosphorylation of 5-FU in the gastrointestinal tissue, reducing the diarrhoea associated with 5-FU.

In a phase II trial of advanced and recurrent SCCHN (59 eligible cases), S-1 showed a high response rate of 28.8% with acceptable toxicities (Inuyama *et al*, 2001). S-1 also showed a higher response in gastric cancer (Sakata *et al*, 1998; Koizumi *et al*, 2000), colorectal cancer (Ohtsu *et al*, 2000), biliary tract cancer (Ueno *et al*, 2004) and so on. In these studies, S-1 was orally administered for 4 weeks followed by a 2-week rest period. Main adverse events were haematological, for example, anemia, leukopenia and neutropenia,, gastrointestinal, for example, anorexia, diarrhoea and nausea, and skin toxicities (Schoffski, 2004). To decrease the toxicity of S-1 without decreasing efficacy and since the median time to the worst toxic events was 22 days for haematological toxicities and 15 days for diarrhoea and stomatitis in the above schedule in advanced gastric cancer (Nagashima *et al*, 2005), we devised the schedule of S-1 for 2-week administration followed by 1-week rest is for adjuvant treatment of patients with locoregionally advanced SCCHN and compared the feasibility of its application with conventional 4-week administration followed by 2-week rest.

## MATERIALS AND METHODS

### Patients

The following eligibility criteria were used: histologically or cytologically confirmed SCCHN, stage III or IV curable disease with no evidence of distant metastases, a primary tumour in the nasopharynx, mesopharynx, hypopharynx, larynx, oral cavity or maxillary sinus, age of 20–74 years, Eastern Cooperative Oncology Group (ECOG) performance status (PS) of 0, 1 or 2, a WBC count of 4000 mm^−3^ or more, an absolute neutrophil count (ANC) of 2000 mm^−3^ or more, a platelet count of 100 000 mm^3^ or more, a haemoglobin level of 9.5 g dl^−1^ or more, AST, ALT and alkaline phosphatase levels below 2.5 times the upper limit of normal (ULN), total bilirubin and creatinine levels below than 1.5 times ULN, a BUN level below the ULN and a 24-h creatinine clearance rate of more than 60 ml min^−1^. The exclusion criteria were patients with previous chemotherapy, radiotherapy or surgery, concomitant malignancy, significant cardiac arrhythmia or heart failure.

In the 4 weeks after the completion of definitive treatments, no clinical evidence of locoregional tumours or distant metastasis were confirmed and adequate organ functions, sufficient oral intake in particular, were evaluated. All patients provided written informed consent prior to enrollment in the study and the protocol was approved by the institutional ethics committee of each participating institution. On entry to the study, the eligibility of patients was checked once more via facsimile by the central administration office (Tokyo).

### Treatment schedule

The randomization was performed centrally at the Division of Environmental and Occupational Health, Toho University School of Medicine, Tokyo. S-1 was administered orally after meals. The dosage of S-1 was selected as follows: in a patient with body surface area (BSA)<1.25 m^2^, 40 mg twice a day (80 mg day^−1^); BSA of 1.25 m^2^ but <1.5 m^2^, 50 mg twice a day (100 mg day^−1^); and BSA⩾1.5 m^2^, 60 mg twice a day (120 mg day^−1^).

Patients receiving definitive treatments were randomly assigned to either arm A, S-1 administration for 2 weeks followed by a 1-week rest or arm B, S-1 administration for 4 weeks followed by a 2-week rest. In both treatment arms, the administration of S-1 was continued for 6 months (eight courses in arm A and four courses in arm B) unless there was any evidence of recurrence, other malignancies or severe adverse events. The planned total administration days of S-1 was 112 days in both arms and the planned total administration doses of S-1 were also identical in both arms.

During the study, the dosage of S-1 was adjusted according to the degree of haematological and nonhaematological toxicities. The dosage was planned to be reduced by one level (20 mg per day) in patients with evidence of grade 3 haematological toxicity, or grade 2 or more nonhaematological toxicity. If recovery from such toxicities was confirmed at a reduced dose, the administration at the reduced dosage was continued. If a patient with BSA<1.25 m^2^ experienced the above toxicities, then no further treatment with S-1 was done. Patients were observed for at least 1 year after initiation of S-1 administration.

### Evaluation of feasibility and toxicity

All eligible patients who had received any definitive treatment were considered assessable for feasibility and toxicity. Feasibility was evaluated by the total administration days and the relative total administration dose of S-1. The number of patients was calculated at the time when S-1 administration was stopped or the planned administration dose per day was reduced because of adverse events of S-1. The total administration days of S-1 (the planned total administration days of S-1, 112 days) was evaluated by the cumulative rate of patients. The relative total administration dose was expressed by the rate between the actual total administration dose and the planned total administration dose, and the cumulative rate of the relative total administration dose was evaluated in each arm. Patients in whom administration of S-1 was halted due to tumour recurrence or other non-S-1-related complications were treated as censored cases. Complete blood count and blood chemistry studies were performed weekly. Adverse events were graded according to the National Cancer Institute-Common Toxicity Criteria (NCI-CTC) version 2.0.

### Statistical analysis

Patient characteristics, feasibility, adverse events, disease-free survival and recurrent sites were analysed. The cumulative total administration days, the cumulative relative total administration dose and the 1-year disease-free survival were examined by the Kaplan–Meier method and the difference in the two arms was calculated by the log-rank test. The differences in the mean total administration days and the mean relative total administration dose between the two arms were compared with Student’s *t*-test. The differences in adverse events were evaluated by the *χ*^2^ test. The data were considered to be significant when the *P*-value was ⩽0.05.

## RESULTS

### Patient characteristics

From April 2002 to December 2003, 102 patients with stage III and IV SCCHN who had received definitive treatments were enrolled and randomized (51 cases in arm A and 51 in arm B) and all patients were eligible. One patient in arm A refused the present study and did not receive chemotherapy or other treatment. The each number of patients (intention-to-treat population) studied was 50 cases in arm A and 51 in arm B.

The patient characteristics showed no statistically significant difference (Table 1). In total, 84% of patients in arm A and 74.5% in arm B had a PS of 0. In total, 68% of patients in arm A and 68.5% in arm B had Stage IVA. The primary therapy had mainly been surgery or concurrent chemoradiation in both arms.

### Feasibility

**Figure 1:**
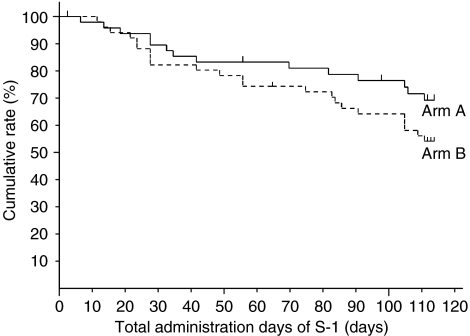
Cumulative rate of total administration days of S-1 in each arm by Kaplan–Meier method; total administration days of S-1 within a 112-day administration period were 69.4% (95% CI: 56.0–82.9%) in arm A and 54.4% (95% CI: 40.6–68.1%) in arm B, respectively.

**Figure 2:**
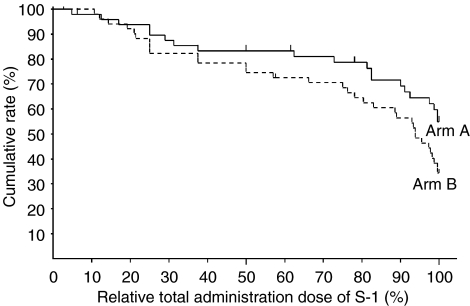
Cumulative rate of relative total administration dose of S-1 in each arm by Kaplan–Meier method; relative total administration dose of S-1 at 100% were 54.9% (95% CI: 40.1–69.7%) in arm A and 34.3% (95% CI: 21.1–47.4%) in arm B (*P*<0.1), respectively.

In total, 40% of patients in arm A receive S-1 administration along the planned schedule and dose, while 29% in arm B did (not significant: NS) (Table 2). The rates of cases that discontinued S-1 administration due to adverse events were 22.0% in arm A and 29.4% in arm B, respectively (Table 2). The cumulative rates of total administration days of S-1 on day 112 were 69.4% (95% CI: 56.0–82.9%) in arm A and 54.4% (95% CI: 40.6–68.1%) in arm B, respectively (NS, *P*=0.147) (Figure 1). The mean of total administration days in each arm was almost identical (87.3 days *vs* 87.2 days) (Table 3). The cumulative rates of the relative total administration dose at 100% were 54.9% (95% CI: 40.1–69.7%) in arm A and 34.3% (95% CI: 21.1–47.4%) in arm B, respectively (*P*=0.054) (Figure 2). The mean of the relative total administration dose in each arm was similar (76.1 *vs* 75.7%) (Table 3).

### Adverse events

Drug-related adverse events are listed in Table 4. Main adverse events were haematological, gastrointestinal and cutaneous symptoms. There was no grade 4 adverse event in either arm. Adverse events were recorded for 41 patients (82.0%) in arm A and 48 patients (94.1%) in arm B (NS, *P*=0.060) and the severe adverse Grade 3 events were observed in seven patients in arm A and in 14 in arm B (NS). In total, 11 patients (22.0%) in arm A and 15 patients (29.4%) in arm B discontinued S-1 administration because of adverse events induced by the agent (NS).

Stomatitis was observed in six cases of arm A and in four of arm B. Diarrhoea, which was one of dose limiting toxicities in the S-1 studies in Western countries, was found in five patients (10.0%) of arm A and in 14 (27.4%) of arm B. Only one in arm A and two in arm B had grade 3 diarrhoea. In the arm A patient, grade 3 diarrhoea appeared after the completion of three courses and further administration of S-1 was stopped. In one arm B patient, diarrhoea was observed during the first course and the administration was stopped for 7 days. Then the reduced dose of S-1, according to the administration schedule, was begun and continued. The other arm B patient with grade 3 diarrhoea had a 3-day rest during the second course and then continued to receive the same dose without reduction, according to the planned schedule.

In the adverse events induced by the S-1 administration, incidences of diarrhoea (10 *vs* 28%; *P*<0.05) and skin toxicities, that is, rash and pigmentation, (18 *vs* 37%; *P*<0.05) were significantly higher in arm B than in arm A. No patient showed hand-foot syndrome.

### Disease-free survival and recurrence

**Figure 3:**
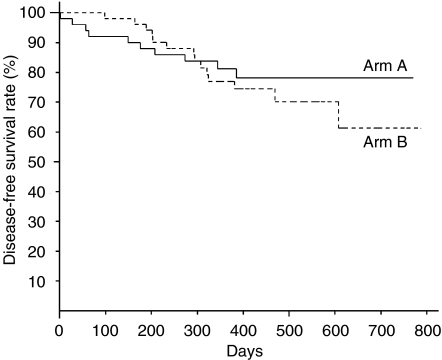
Disease-free survival rate in each arm; 1-year disease-free survival rates were 81.2% (95% CI: 70.0–92.4%) in arm A and 77.0% (95% CI: 65.0–89.0%) in arm B, respectively.

The median follow-up time was 14.7. months (range 12.0–26.2 months). The 1-year disease-free survival rates were 81.2% (95% CI; 70.0–92.4%) in arm A and 77.0% (95% CI; 65.0–89.0%) in arm B, respectively (Figure 3). There was no statistically significant difference in the 1-year disease-free survival in favour of arm A.

In total, 10 patients (20.0%) in arm A and 14 patients (27.5%) in arm B relapsed. Locoregional recurrence was predominant in both arms nine of 10 relapsed patients in arm A and 13 of 14 in arm B. In terms of the site of distant metastasis, there were three cases in arm A, that is, bone (one case) and lung (2 cases), while five cases in arm B showed distant metastases in the lung (3), liver (1) and mediastinum (1).

## DISCUSSION

Recently the Radiation Therapy Oncology Group reported postoperative concurrent radiotherapy and chemotherapy for high-risk SCCHN improved the local and regional control, and disease-free survival (Cooper *et al*, 2004). Adjuvant chemotherapy can also potentially improve the outcome. The regimen of adjuvant chemotherapy in randomized studies consisted of three courses of intravenous low-dose CDDP-based regimen every 6 weeks (Ervin *et al*, 1987), monthly intravenous CDDP (80 mg m^−2^) alone for 6 months (Jacobs and Makuch, 1990), three courses of intravenous CDDP (100 mg m^−2^) with 5-day continuous infusion of 5-FU at 1000 mg m^−2^ day^−1^ (Laramore *et al*, 1992) and 18 courses of intravenous methotrexate (250 mg m^−2^) followed by 5-FU (600 mg m^−2^) with leucovorin rescue over 6 months on an outpatient basis (Johnson *et al*, 1996). A few studies suggested survival benefits (Ervin *et al*, 1987; Johnson *et al*, 1996) and a decrease in the incidence of distant metastases with adjuvant chemotherapy (Jacobs and Makuch, 1990; Laramore *et al*, 1992). The Head and Neck Contracts Study group showed that adjuvant chemotherapy, while reducing distant metastases, does not improve survival significantly in the total of cases studied; however, a significant survival advantage with adjuvant chemotherapy was detected for oral cavity cancer and for N2 disease using a subset analysis (Jacobs and Makuch, 1990). Based on these results, the group concluded there may be particular sites and stages for which adjuvant chemotherapy would be advantageous, since head and neck cancer patients are a heterogeneous group. On the other hand, one reason why adjuvant chemotherapy for SCCHN with no clinically residual tumours after definitive treatments had no impact on survival benefit has been suggested to be the lack of antitumour effect of the adjuvant chemotherapy regimen or the shortage of the duration of adjuvant chemotherapy, or both.

Adjuvant chemotherapy with oral chemotherapeutic agents has not been studied in Western countries for HNSCC. Oral administration of UFT (tegafur and uracil in a 1 : 4 molar concentration) has been shown significant survival benefit in the adjuvant setting in a variety of carcinomas, for example, colorectal, gastric, lung and breast cancer (Akasu *et al*, 2004; Kato *et al*, 2004; Kinoshita *et al*, 2005; Noguchi *et al*, 2005), while 1-year administration of UFT (300 mg day^−1^) as adjuvant chemotherapy for SCCHN after curative surgical treatment significantly prevented distant metastasis with a small survival benefit (Tsukuda *et al*, 1994).

Since S-1 is considered to be a more effective drug than UFT, long-term S-1 administration could be a candidate for adjuvant chemotherapy of SCCHN. However, because S-1 is more toxic than UFT and also the influence of prior therapy must not be ignored in adjuvant chemotherapy, we wondered if the standard schedule of S-1 could be adjusted for long-term administration. In fact, the rate of adverse reactions in gastric cancer patients receiving S-1 adjuvant chemotherapy was higher than in phase II studies (Kinoshita *et al*, 2004).

From the viewpoint of the long-term administration for over 6 months in the adjuvant chemotherapy setting, we considered that a treatment schedule of 2-week administration followed by a 1-week rest might be more feasible and safer with the same antitumour effects as those with the conventional 4-week administration followed by 2-week rest, since the dose intensity is identical, but the rest time is spread out in the former method. Furthermore, the results of phase I and II trials using combined CDDP with S-1, that is, administration of S-1 at 40, 50 or 60 mg m^−2^ twice a day for 14 consecutive days and CDDP at 60 or 70 mg m^−2^ on the mid-day of S-1 administration (Fujii *et al*, 2005) encouraged us to conducted the current feasibility study of different administration schedules.

The rate of cases which discontinued S-1 administration due to adverse reactions was low in arm A than in arm B and the cumulative rate of total administration days of S-1 by day 112 (6 months after starting administration) in arm A was higher (69.4%) than that in arm B (54.4%). Furthermore, the cumulative rate of relative total administration dose calculated by the rate between the actual total administration dose and planned total administration dose of S-1 was higher in arm A (54.9%) than arm B (34.3%). The completion rate in patients who received the planned schedule were not enough; however, the mean of the relative total administration dose was 76.1% and more than 70% of the patients could be administered eight courses in arm A. Adverse events were recorded for 41 patients (82.0%) in arm A and 48 patients (94.1%) in arm B, most of which were haematological, gastrointestinal and skin toxicities, as in the phase II study. Most of adverse events in grade 1 or 2 were controllable. Since S-1 was administered consecutively, most of the adverse events, such as neutropenia, diarrhoea and pigmentation, were getting worse gradually. So we thought that in both arms the rest periods prevented the aggravation of most adverse events and kept them in low grades. However, arm B, the longer consecutive administration of S-1 caused the higher incidence and grade of adverse events compared with arm A. Especially, the incidences of diarrhoea (10 *vs* 28%; *P*<0.05) and skin toxicity (18 *vs* 37%; *P*<0.05) were significantly higher in arm B than in arm A. The 1-year disease-free survival rates were 81.2% in arm A and 77.0% in arm B, with no statistically significant difference.

These results suggest that 2-week administration followed by 1-week rest seems to be more tolerable and safer compared to 4-week administration followed by a 2-week rest. Since higher incidence and more severe degree of diarrhoea have been found in Western countries during S-1 administration, the 2-weeks course holds potential for therapeutic applications. Owing to the short-term follow-up in the current study, the potential to reduce relapse after definitive treatments in advanced but curable SCCHN should be carefully examined in the future. In terms of adjuvant chemotherapy, we are planning the phase III comparison study between S-1 with 2-week administration followed by 1-week rest and daily UFT administration for 1 year in locoregionally advanced SCCHN.

## Figures and Tables

**Table 1:** Patient characteristics

	**Arm A (*n*=50)**		**Arm B (*n*=51)**	
**Characteristics**	**No.**	**%**	**No.**	**%**
*Gender*				
Male	44	88	48	94.1
Female	6	12	3	5.9
				
*Age (years)*				
Median	55.5		60.0	
(Range)	(25–73)		(35–74)	
		
*PS*				
0	42	84	38	74.5
1	7	14	12	23.5
2	2	4	1	2.0
				
*Primary sites*				
Maxillary sinus	5	10	6	11.8
Oral cavity	8	16	14	27.5
Oropharynx	2	4	1	2.0
Nasopharynx	14	28	8	15.7
Hypopharynx	11	22	16	31.4
Larynx	10	20	6	11.8
				
*Stage*				
III	14	28	13	25.5
IV-A	34	68	35	68.6
IV-B	2	4	3	5.9
				
*Primary therapy*				
Surgery	22	44	24	47.1
Radiotherapy alone	5	10	4	7.8
Concurrent chemoradiation	23	46	23	45.1

**Table 2:** Administration of S-1

	**Arm A (*n*=50)**		**Arm B (*n*=51)**	
	**No.**	**%**	**No.**	**%**
Patients following planned schedule and dose	20	40.0	15	29.4
Patients in whom administration was halted due to adverse events	11	22.0	15	29.4
Patients with dose-reduction	7	14.0	6	11.8
Patients with delayed courses	14	28.0	19	37.3

**Table 3:** Feasibility of S-1

	**Arm A (*n*=50)**	**Arm B (*n*=51)**
*Cumulative rate of total administration*		
days of S-1 on 112-day (95% CI)	69.4% (56.0–82.9%)	54.4% (40.6–68.1%)
		
*Total administration days*		
Mean	87.3 days	87.2 days
Standard deviation	37.3 days	35.7 days
		
*Cumulative rate of the relative total*		
administration dose of S-1 at 100% (95% CI)	54.9% (40.1–69.7%)	34.3% (21.1–47.4%)
		
*Relative total administration dose*		
Mean	76.1%	75.7%
Standard deviation	33.2%	31.3%

**Table 4:** Drug-related adverse events

	**Arm A (*n*=50)**		**Arm B (*n*=51)**	
	**G1/2**	**G3**	**G1/2**	**G3**
	**No. (%)**	**No. (%)**	**No. (%)**	**No. (%)**
Leukopenia	17 (34)	3 (6)	20 (39)	3 (6)
Neutropenia	12 (24)		11 (22)	4 (8)
Thrombocytepenia	9 (18)		9 (18)	
Anaemia	13 (26)		17 (33)	1 (2)
Elevation of Bilirubin	7 (14)		7 (14)	2 (4)
Elevation of ALT, AST	7 (14)		8 (16)	1 (2)
Anorexia	14 (28)		14 (27)	
Nausea and vomiting	7 (14)		15 (29)	
Diarrhoea	4 (8)	1 (2)	12 (24)	2 (4)
Stomatitis	6 (12)		3 (6)	1 (2)
Other GI toxicity	3 (6)		2 (4)	
Cutaneous symptoms	9 (18)		19 (37)	
Peripheral neuropathy	1 (2)		2 (4)	
Dizziness	2 (4)	1 (2)	2 (4)	
General fatigue	10 (20)		6 (12)	
Others	8 (16)		2 (4)	
